# Efficiency and Workload Reduction of Semi-automated Citation Screening Software for Creating Clinical Practice Guidelines: A Prospective Observational Study

**DOI:** 10.2188/jea.JE20230227

**Published:** 2024-08-05

**Authors:** Takehiko Oami, Yohei Okada, Masaaki Sakuraya, Tatsuma Fukuda, Nobuaki Shime, Taka-aki Nakada

**Affiliations:** 1 Department of Emergency and Critical Care Medicine, Chiba University Graduate School of Medicine, Chiba, Japan; 2 Department of Preventive Services, Kyoto University Graduate School of Medicine, Kyoto, Japan; 3 Health Services and Systems Research, Duke-NUS Medical School, National University of Singapore, Singapore, Singapore; 4 Department of Emergency and Intensive Care Medicine, JA Hiroshima General Hospital, Hiroshima, Japan; 5 Department of Emergency and Critical Care Medicine, Toranomon Hospital, Tokyo, Japan; 6 Department of Emergency and Critical Care Medicine, Graduate School of Biomedical and Health Sciences, Hiroshima University, Hiroshima, Japan

**Keywords:** citation screening, systematic review, sepsis, clinical practice guidelines

## Abstract

**Background:**

We evaluated the applicability of automated citation screening in developing clinical practice guidelines.

**Methods:**

We prospectively compared the efficiency of citation screening between the conventional (Rayyan) and semi-automated (ASReview software) methods. We searched the literature for five clinical questions (CQs) in the development of the Japanese Clinical Practice Guidelines for the Management of Sepsis and Septic Shock. Objective measurements of the time required to complete citation screening were recorded. Following the first screening round, in the primary analysis, the sensitivity, specificity, positive predictive value, and overall screening time were calculated for both procedures using the semi-automated tool as index and the results of the conventional method as standard reference. In the secondary analysis, the same parameters were compared between the two procedures using the final list of included studies after the second screening session as standard reference.

**Results:**

Among the five CQs after the first screening session, the highest and lowest sensitivity, specificity, and positive predictive values were 0.241 and 0.795; 0.991 and 1.000; and 0.482 and 0.929, respectively. In the secondary analysis, the highest sensitivity and specificity in the semi-automated citation screening were 1.000 and 0.997, respectively. The overall screening time per 100 studies was significantly shorter with semi-automated than with conventional citation screening.

**Conclusion:**

The potential advantages of the semi-automated method (shorter screening time and higher discriminatory rate for the final list of studies) warrant further validation.

## INTRODUCTION

A systematic literature review is a critical component in developing clinical practice guidelines, collecting and synthesizing all available evidence on a topic.^1^ It is time-consuming and labor-intensive, since numerous publications must be screened.^2^^,^^3^ Various software programs employing automation techniques have emerged to aid citation screening by leveraging predefined datasets.^4^^–^^8^ Although the application of such software is still in its infancy, it holds promise for substantially enhancing the efficiency and effectiveness of systematic reviews.^9^

Although machine learning-based automated screening has various advantages, its application in systematic reviews for guideline development remains limited. Potential barriers to its use include lack of trust, difficulty in implementation, inappropriate software selection, incompatibility with conventional methods, and variable usability across different search fields.^9^^,^^10^ These concerns can be addressed through diligent research and application; however, the feasibility and validity of these software programs in the context of clinical practice guideline development has not been elucidated. Therefore, evaluating its efficacy for citation screening in the context of guideline formulation is crucial.

Since clinical practice guidelines usually have numerous clinical questions (CQs) that require extensive systematic reviews, well-performed semi-automated screening tools would offer advantages over conventional methods. We hypothesized that semi-automated citation screening tools would exhibit sufficient quality and alleviate the workload involved in developing clinical practice guidelines. Therefore, we conducted a comparative analysis to examine the efficiency and reduction in workload associated with the implementation of semi-automated software, opposed to manual literature screening, using the CQs of the Japanese Clinical Practice Guidelines for Management of Sepsis and Septic Shock (J-SSCG).

## METHODS

### Study design and process

We conducted a prospective study to validate the efficiency of semi-automated screening software for the development of the J-SSCG 2024, including the following steps:

1. Selection of citation screening tools suitable for implementation by committee members
2. Definition of clinical questions for investigation
3. Independent performance of citation screening to evaluate the semi-automated citation screening tool in comparison with the conventional method

The review protocol was submitted to a preprint server (medRxiv: DOI:10.1101/2022.11.17.22282374).

### Clinical questions in the J-SSCG

The Japanese Society of Intensive Care Medicine (JSICM) and the Japanese Association for Acute Medicine (JAAM) released the J-SSCG 2020 in 2020. These guidelines incorporated unique clinical conditions and requirements specific to Japan for sepsis and septic shock.^11^ An updated version of the J-SSCG 2020, the J-SSCG 2024, is scheduled for publication in 2024.

We assessed the efficacy of automated software used in a systematic review conducted for the newly identified CQs outlined in the guidelines. The working group members undertook comprehensive literature searches across CENTRAL, PubMed, and Ichushi-Web after establishing a meticulous search strategy and ensuring inclusion of all key studies. Only publications in Japanese or English were included in this study. Subsequently, the team collected all titles and abstracts, integrated the files obtained from various search engines, and removed duplicate files using citation manager software. For the J-SSCG 2024, EndNote (Clarivate Analytics, Philadelphia, PA, USA) was used as the citation manager software. We selected five specific CQs related to initial resuscitation/inotropes as the primary focus of the investigation, aligning the timing of conventional citation screening with the progression of this study (eTable 1).

### Semi-automated citation screening

To determine the optimal automated citation-screening software, we used the following criteria:

1. Open-source software with high reproducibility and applicability for systematic review
2. Software developed using machine learning techniques

Consequently, four software programs were selected: ASReview,^4^ Colandr (https://www.colandrcommunity.com/), FASTREAD (https://github.com/fastread/src), and RobotReviewer.^12^ We evaluated the user-friendliness and compatibility of the semi-automated software with other systematic review tools, taking into account the ease of use for the members of the J-SSCG working group (eFigure 1). Although Colandr can be used as a web application, its slow operation is a potential barrier to reducing the workload. FASTREAD has no function to import files from EndNote. As RobotReviewer requires command line input for installation and a part of the process, its complex workload could be a disturbance to members of the working group. Therefore, RobotReviewer may not be optimal software due to its low generalizability. Finally, we selected ASReview^4^ as the semi-automated citation screening software to evaluate its efficiency and feasibility for guideline development.

ASReview is an open-source machine learning software using active learning for systematic reviews. To use the software, the reviewers initially uploaded a dataset of references from the citation manager. Subsequently, the software was required to tag at least one relevant study and create a list of non-relevant references to be tagged by the reviewers. In this study, as relevant references, we uploaded all the key studies—those expected to be selected by the guideline members.^13^ As the time to stop the screening process was at the discretion of the reviewer, we opted to tag at least 100 randomly selected references. Based on these training data, the software employed machine learning techniques to determine the relevance of the remaining publications. The active learning model updates the order of records to be screened based on the labelled records and reduces the number of manually screened records during the screening process. While ASReview implements several classifiers, feature extraction techniques, query strategies, and balance strategies, we selected the default setup (Term Frequency–Inverse Document Frequency, Naive Bayes, Maximum, Dynamic resampling), which is recommended given its speed and excellent performance. The software then identified the relevant studies for each CQ and generated a file containing the results. Two independent reviewers (T.O. and Y.O.) who were not involved in the development of the J-SSCG 2024 performed simultaneous semi-automated citation screening using the software. Each reviewer received references deemed relevant, based on the decision of the software. Following the screening session, the two reviewers discussed the included studies and resolved any conflicts in the results until a consensus was reached. The session using the semi-automated software only reviewed the first screening for the research topic.

### Conventional citation screening

The files processed in EndNote were imported into Rayyan (https://rayyan.qcri.org/welcome) software designed to facilitate systematic reviews.^14^ At least two independent reviewers assessed the relevance of each publication based on the title and abstract. In cases of disagreement, the reviewers discussed the judgement to reach a consensus. If a disagreement could not be resolved, a third person conducted the review. After the first screening of the title and abstract, a second screening was performed using the full text of the articles.

### Data collection

We collected data on various reviewer characteristics, including age, sex, professional category, educational degree, field of specialty, duration of clinical experience, and total number of completed systematic reviews. We compared the accuracy and workload of conventional literature screening with those of the semi-automated software. Accuracy was assessed by counting the number of missing or redundant references after the first screening session between conventional and semi-automated procedures. The workload was evaluated by documenting the total duration of the citation screening session. For ASReview, the reviewers recorded a video of their citation screening session. To ensure objectivity, two independent reviewers (T.O. and Y.O.) measured the total time elapsed from the start of the review to the end of the session. The time taken by users to label and upload data to ASReview was not assessed. As Rayyan provides a built-in function for time measurement during literature screening, we recorded the duration from the beginning of the screening to the end of the session. During this study, systematic reviews of the new CQs of the J-SSCG 2024 had not yet been conducted.

### Statistical analyses

**Figure 1.**
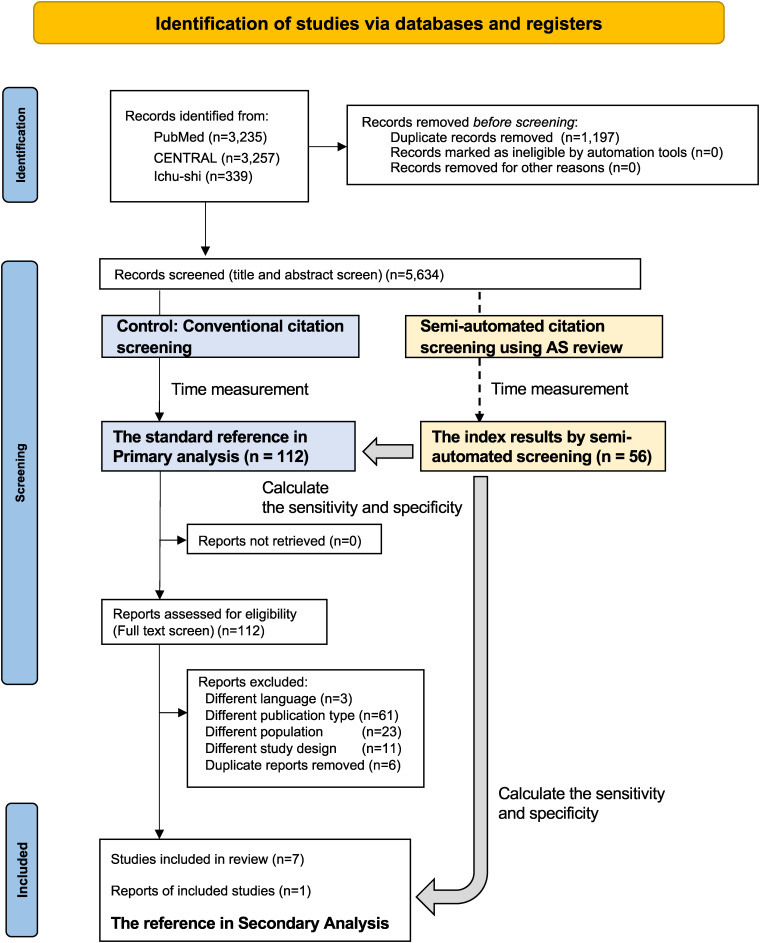
Schematic chart of the systematic review of clinical questions using semi-automated citation screening and conventional citation screening. A flow diagram of the systematic review through identification, first screening, and second screening for Clinical Question 1 is presented. The timing of the evaluation of the accuracy and screening time between the semi-automated screening and the conventional method in the primary and secondary analyses is depicted with the screening process on the diagram.

We planned the study to minimize the risk of bias by referring to the QUADAS-2 tool^15^ and registered the protocol before starting the analysis, as mentioned above. To compare the accuracy and workload of the semi-automated software with those of the conventional manual screening method, we set the results of the conventional method after the first screening session as the reference in the primary analysis. The index results were those of the automated screening. We calculated the sensitivity, specificity, and positive predictive value (PPV) of the automated software by comparison with the number of references extracted through manual screening. We also normalized the screening time based on the number of publications screened for each CQ and compared the session times between the two groups. Furthermore, we set the reference value as the result of the conventional method after secondary screening in the secondary analysis. Subsequently, we compared the accuracy of the semi-automated software with that of manual screening following the first round of screening. As our initial aim was to assess the feasibility of the software during the first screening session at the time of protocol registration, this secondary analysis was not designated as a pre-planned analysis (Figure 1). Following the completion of the primary analysis, we performed a post-hoc sensitivity analysis to explore whether alterations in the threshold would impact the sensitivity and specificity. In the absence of an objectively established threshold for determining the relevance or irrelevance of references, we set the number of tagged references to be screened to 200 and implemented the screening process in the same manner.

Continuous variables are summarized as mean (standard deviation) or median (interquartile range [IQR]), as appropriate. The sensitivity, specificity, and PPVs are presented with 95% confidence intervals (CIs). The selection of a statistical test, either an unpaired *t*-test or the Mann–Whitney *U* test, was based on the normality of the distribution. A *P*-value of less than 0.05 was considered statistically significant. Statistical analyses were performed using the GraphPad Prism 9 software (GraphPad Software, San Diego, CA, USA).

## RESULTS

### Reviewers’ characteristics in the study

Two reviewers conducted literature screening using the semi-automated software, whereas 18 reviewers from the J-SSCG 2024 Working Group performed conventional citation screening. The reviewers had a median age of 38 years (range: 35–40) and the majority (95%, 19/20) were males. Most were medical doctors (95%, 19/20) with specialties in emergency medicine and/or critical care. Most had no prior experience with systematic reviews (80%, 16/20). The characteristics of the reviewers are detailed in Table 1.

**Table 1.** Reviewers’ characteristics in the citation screening

	Total	Semi-automated citation screening	Conventional citation screening
Number of reviewers	20	2	18

Age, years	38.0 (35–40)	39.5 (38–41)	38.0 (35–39)
Male sex, *n* (%)	19 (95.0)	2 (100)	17 (94.4)
Profession			
Medical doctor, *n* (%)	19 (95.0)	2 (100)	17 (94.4)
Nurse, *n* (%)	1 (5.0)	0 (0)	1 (5.6)
Specialty^a^			
Emergency medicine, *n* (%)	14 (70.0)	2 (100)	12 (66.7)
Critical care, *n* (%)	16 (80.0)	2 (100)	14 (77.8)
Anesthesiology, *n* (%)	4 (20.0)	0 (0)	4 (22.2)
Others	3 (15.0)	0 (0)	3 (16.7)
Doctor of Philosophy, *n* (%)	9 (45.0)	2 (100.0)	7 (38.9)
Length of clinical experience			
0–5 years, *n* (%)	0 (0)	0 (0)	0 (0)
6–10 years, *n* (%)	5 (25.0)	0 (0)	5 (27.8)
11–15 years, *n* (%)	10 (50.0)	1 (50.0)	9 (50.0)
16–20 years, *n* (%)	5 (25.0)	1 (50.0)	4 (22.2)
≥21 years, *n* (%)	0 (0)	0 (0)	0 (0)
Number of systematic review publications			
None, *n* (%)	16 (80.0)	1 (50.0)	15 (83.3)
1–5 papers, *n* (%)	3 (15.0)	0 (0)	3 (16.7)
6–10 papers, *n* (%)	0 (0)	0 (0)	0 (0)
≥11 papers, *n* (%)	1 (5.0)	1 (50.0)	0 (0)

Data are presented as median and interquartile range for continuous variables.

^a^The category of specialty includes multiple inputs.

### Literature selection process

The study flowchart is shown in Figure 1 and eFigure 2. In total, 26,759 publications were extracted through a comprehensive literature search targeting five CQs. Specifically, 5,634 publications were included in CQ1, 3,418 in CQ2, 1,038 in CQ3, 4,326 in CQ4, and 2,253 in CQ5, after removing duplicates during the first screening process.

In the conventional groups, literature screening was conducted using the title and abstract reading method on the Rayyan platform. Consequently, 112 publications were selected for full-text screening for CQ1, 17 for CQ2, 14 for CQ3, 70 for CQ4, and 39 for CQ5. We set this result as the standard reference for the primary analysis. The median screening time per 100 studies for each individual during the first screening was 14.7 minutes (minimum, 13.1; maximum, 18.7 min) for CQ1, 11.9 minutes (minimum, 10.0; maximum, 31.1 min) for CQ2, 16.3 minutes (minimum, 7.9; maximum 31.2 min) for CQ3, 15.8 minutes (minimum, 13.1; maximum, 27.7 min) for CQ4, and 11.7 minutes [minimum, 10.5; maximum, 17.1 min] for CQ5. Subsequently, 41 publications (8 in CQ1, 4 in CQ2, 4 in CQ3, 17 in CQ4, and 8 in CQ5) were selected for qualitative analysis during the secondary full-text screening in each systematic review. We set this result as the standard reference for the secondary analysis.

In the group that used the semi-automated application, literature screening was conducted by reading titles and abstracts using ASReview. Consequently, 56 publications were selected for CQ1, 14 for CQ2, 14 for CQ3, 40 for CQ4, and 50 for CQ5. The median screening time per study for each individual in the groups was 0.8 minutes (minimum, 0.5; maximum, 1.1 min) for CQ1, 1.2 minutes (minimum, 1.0; maximum, 1.4 min) for CQ2, 3.4 minutes (minimum, 2.3; maximum, 4.5 min) for CQ3, 1.3 minutes (minimum, 1.2; maximum, 1.3 min) for CQ4, and 1.5 minutes (minimum, 1.1; maximum, 1.9 min) for CQ5. The number of included studies for the qualitative analysis, following semi-automated citation screening, was seven for CQ1, four for CQ2, three for CQ3, 16 for CQ4, and eight for CQ5.

### Accuracy of the semi-automated citation screening for literature review

Among the five CQs, the sensitivity and specificity of the index results of semi-automated screening were 0.241 and 0.995 for CQ1, 0.765 and 1.000 for CQ2, 0.786 and 0.997 for CQ3, 0.400 and 0.997 for CQ4, and 0.795 and 0.991 for CQ5, respectively, in the primary analysis (Table 2).

**Table 2.** Accuracy of the semi-automated citation screening software compared with the conventional method

	TN	FP	FN	TP	Sensitivity	Specificity	Positive predictive value
CQ1	5,493	29	85	27	0.241 (0.171–0.328)	0.995 (0.993–0.996)	0.482 (0.357–0.610)
CQ2	3,400	1	4	13	0.765 (0.527–0.904)	1.000 (0.998–1.000)	0.929 (0.685–0.996)
CQ3	1,021	3	3	11	0.786 (0.524–0.924)	0.997 (0.991–0.999)	0.786 (0.524–0.924)
CQ4	4,244	12	42	28	0.400 (0.293–0.517)	0.997 (0.995–0.998)	0.700 (0.546–0.819)
CQ5	2,195	19	8	31	0.795 (0.645–0.892)	0.991 (0.987–0.995)	0.620 (0.482–0.741)

CQ, clinical question; FN, false negative; FP, false positive; TN, true negative; TP, true positive.

The sensitivity, specificity, and positive predictive values are presented with 95% confidence intervals.

Next, we used the final list of included studies following the second screening, with conventional screening as the standard reference, and compared the accuracy of the two methods in the secondary analysis. Most studies screened with the semi-automated software were included in the final list of studies for the qualitative analysis. While the sensitivity of automatic citation screening ranged from 0.750 to 1.0, the specificity and PPVs were comparable between the two methods (Table 3 and eTable 2).

**Table 3.** Comparison of statistics on sensitivity, specificity, and predictive value using the included studies for the qualitative analysis^a^

	TN	FP	FN	TP	Sensitivity	Specificity	Positive predictive value
Semi-automated citation screening							

CQ1	5,577	49	1	7	0.875 (0.529–0.994)	0.991 (0.989–0.993)	0.125 (0.062–0.236)
CQ2	3,404	10	0	4	1.000 (0.510–1.000)	0.997 (0.995–0.998)	0.286 (0.117–0.547)
CQ3	1,023	11	1	3	0.750 (0.301–0.987)	0.989 (0.981–0.994)	0.214 (0.076–0.476)
CQ4	4,285	24	1	16	0.941 (0.730–0.997)	0.994 (0.992–0.996)	0.400 (0.264–0.554)
CQ5	2,203	42	0	8	1.000 (0.676–1.000)	0.981 (0.975–0.986)	0.160 (0.083–0.285)

Conventional citation screening							

CQ1	5,522	104	0	8	1.000 (0.676–1.000)	0.982 (0.977–0.985)	0.071 (0.037–0.135)
CQ2	3,401	13	0	4	1.000 (0.510–1.000)	0.996 (0.994–0.998)	0.235 (0.096–0.473)
CQ3	1,024	10	0	4	1.000 (0.510–1.000)	0.990 (0.982–0.995)	0.286 (0.117–0.547)
CQ4	4,256	53	0	17	1.000 (0.816–1.000)	0.988 (0.984–0.990)	0.243 (0.158–0.355)
CQ5	2,214	31	0	8	1.000 (0.676–1.000)	0.986 (0.981–0.990)	0.205 (0.108–0.355)

CQ, clinical question; FN, false negative; FP, false positive; TN, true negative; TP, true positive.

The sensitivity, specificity, and positive predictive values are presented with 95% confidence intervals.

^a^The final list of the included studies for the qualitative analysis was set as the standard reference.

### Comparison of overall citation screening time for 100 studies between the automatic and conventional methods

**Figure 2.**
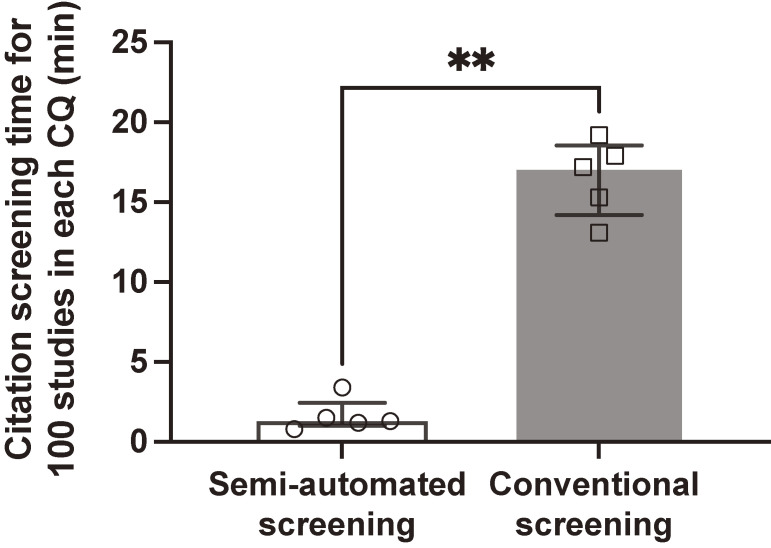
Comparison of the citation screening time for 100 studies between the semi-automated and conventional methods. The difference in processing time was −15.90 min (95% confidence interval, −17.90 to −11.80, *P* = 0.008). The Mann–Whitney *U* test was used for the analysis.

After normalizing the screening time in each CQ with the number of screened studies (eTable 3), we found a significantly shorter overall processing time for 100 studies associated with the semi-automated screening method (1.3; IQR, 1.0–2.5 min) than with the conventional screening method (17.2; IQR, 14.2–18.6 min) using the Mann–Whitney *U* test (mean difference −15.90 min; 95% CI, −17.90 to −11.80; *P* = 0.008) (Figure 2).

### Sensitivity analysis

In the post-hoc sensitivity analysis aimed at changing the threshold by increasing the number of tagged references from 100 to at least 200, the sensitivity of the semi-automated method in CQ1 increased to 0.50 in the primary analysis and 1.000 in the secondary analysis (eTable 4).

## DISCUSSION

With advancements in artificial intelligence, automated citation screening has been implemented in systematic reviews. Here, we demonstrated that the sensitivity and specificity of the semi-automated software were 0.241–0.795 and 0.991–1.000, respectively, with the studies included through conventional citation screening during the first screening session as the standard reference. Additionally, the semi-automated screening method successfully discriminated the included studies after the second screening session with the conventional screening method. Furthermore, the processing time of the semi-automated citation screening method was significantly shorter than that of the conventional method.

Previous studies demonstrated the high accuracy of a semi-automated systematic review, indicating semi-automation as a promising technology.^16^^–^^18^ Other publications employing similar software have indicated the feasibility of replacing the conventional method with an efficient automated approach for screening studies in a systematic review.^19^^,^^20^ Among several software packages that use natural language processing tools, the reliance on prior training data input varies.^6^^,^^9^ For instance, DistillerAI requires a training dataset comprising 40 excluded and 10 included references for active machine learning.^9^ Conversely, ASReview, used in this study, requires only one relevant study and five irrelevant studies to execute a prediction model.^4^ While some machine learning screening tools make predictions based on the probability of the included studies,^21^ ASReview updates the predictions and reorders the ranking during the screening process. This approach, “continuous machine training and ranking,” is recommended in the recent guidelines and some reviews. The question that remains in terms of using ASReview is when to stop the screening process. ASReview suggests several stopping criteria, including time-based, data-based, and mixed strategies; however, the definitive superior strategy remains to be determined. As the accuracy of these tools is contingent on the size of the training data, the relationship between the quantity of input data and software efficiency should be examined in future research.

The accuracy of the software in distinguishing relevant publications also varies. In a previous study using Abstrackr, the sensitivity of the software compared to human decision exceeded 0.75^16^; however, the accuracy relied on the screening assignment. An alternative inquiry utilizing another natural language processing tool, Covidence, reported sensitivity and PPVs of 0.90 and 0.92, respectively.^19^ Here, we demonstrated that the sensitivity of the automated citation screening in the primary analysis varied from 0.20 to 0.75, which is lower than that found in the previous study that investigated the accuracy of ASReview.^4^ The difference can be explained by the setting of the standard reference: whereas the previous report set the final list of included studies as the standard reference, we used the results of the conventional method after the first screening as the standard reference in the primary analysis. Since the sensitivity or PPV depends on the characteristics of the reviewers, obtaining consistent results between the two methods in the primary analysis might be challenging. Conversely, the sensitivity and PPVs achieved better precision when we set the final list of included studies for qualitative analysis as a reference in the secondary analysis. Approximately 80% of the studies were excluded from the final list. For the CQ with the lowest sensitivity after the first semi-automated screening, the PPV using the conventional method was lower than that of the other CQs, indicating that the included list of studies after the first screening with the conventional method included numerous irrelevant studies. Another concern with semi-automated citation screening is its inconsistent accuracy. In a previous study investigating the accuracy of automated software, accuracy varied among different specialties.^9^ Future research should focus on other areas to validate automated software.

A consistent advantage of using automated citation screening software is a reduced review process workload, although the extent of the reduction varies in previous studies. One study demonstrated a 45.9% workload reduction compared with the conventional approach.^19^ Here, we demonstrated a reduction of over 90%. This result may be dependent on the number of studies screened to provide training data in the semi-automated screening software. As the number of studies screened for training data increases, the accuracy of the model may improve, albeit at the expense of prolonged screening time in the semi-automated citation method. The optimal balance between accuracy and the number of publications screened should be determined based on the required quality of the project.

To our knowledge, few clinical practice guidelines have employed semi-automated citation screening methods during the development process. Since clinical practice guidelines usually have numerous CQs that require extensive systematic reviews, well-performed semi-automated screening tools would offer advantages over conventional methods. Another feature of guideline development that differs from the usual systematic review is the limited timeframe available for content publication. Within this constrained timeline, semi-automated citation screening could reshape the framework of clinical practice guidelines. However, we need to be cautious regarding blindly depending on the software. Here, we missed some pivotal articles that comprised the final list of included studies for qualitative analysis, including one published in 1995 that potentially used different terminology or definitions than those used in the current studies. Two other studies were post-hoc or secondary analyses of the primary cohort. To avoid underestimation, it is essential to establish clear and valid criteria for inclusion based on the features of the semi-automated screening software. Additionally, meticulous searches of the key articles, an increased number of tagged references to evaluate relevant or irrelevant for the purpose of training, and hybrid screening (including conventional and semi-automated methods) would cover the overlooked studies in the semi-automated screening process. Since only one study had potentially greater weight than the other studies, the degree of tolerance for missed studies should be determined before the session.

This study had several limitations. First, the number of CQs was limited; thus, overestimation of the accuracy of semi-automated citation screening is possible. Second, we excluded certain software due to lack of applicability in clinical practice guidelines. Alternative automation tools can offer greater accuracy and efficiency in various situations. Third, we demonstrated the high accuracy of the semi-automated screening tool using the results of the included references for qualitative analysis. However, this was not originally planned as part of the analysis. Therefore, the results should be interpreted with caution. Fourth, the study scope was limited to critical care and emergency medicine. When applying the findings to other specialties, caution should be exercised when using a semi-automated citation screening tool. Fifth, the reviewer characteristics displayed heterogeneity within the scope of this study, suggesting that differences in competence and expertise may have resulted in disparate conclusions. For example, the overrepresentation of individuals with limited systematic review experience in the conventional method might have led to an overestimation. Finally, although we incorporated some CQs that constitute clinical practice guidelines, the screening results obtained from the semi-automated tool were not employed in the creation of the guidelines. Therefore, the efficiency of semi-automated screening tools in the guideline development process remains undetermined. Future research should explore the feasibility and validity of using semi-automated software for guideline development.

In conclusion, this study demonstrated that the sensitivity and specificity of the semi-automated software ranged from 0.241 to 0.795 and 0.991 to 1.000, respectively, using the references as studies included in the conventional method in the first screening process. Moreover, the software provided a shorter screening time and identified key studies for the development of clinical practice guidelines. Further research is warranted to validate the efficiency of automation tools.

## SUPPLEMENTARY MATERIAL

eTable 1. The list of the patient/population/problem, intervention, comparison, and outcomes of the selected clinical questions

eTable 2. The list of unidentified studies using the semi-automated citation screening software for the qualitative analysis

eTable 3. Comparison of citation screening time between the semi-automated and conventional methods

eTable 4. Accuracy of the semi-automated citation screening software with an increased number of tagged references

eFigure 1. Flowchart of the software selection in this study

eFigure 2. PRISMA 2020 flow diagram of the systematic review of clinical questions

A flow diagram of the systematic review through identification and first screening for clinical questions (CQ) (from CQ2 to CQ5) is shown.
