# Supplementary material for: Efficiency and Workload Reduction of Semi-automated Citation Screening Software for Creating Clinical Practice Guidelines: A Prospective Observational Study
Source: J Epidemiol. 2024 Aug 5;34(8):380–6. doi: 10.2188/jea.JE20230227 (PMC11230876; doi:10.2188/jea.JE20230227)
Supplement: Supplementary file 1 [file je-34-380-s001.pdf]

eTable 1. The list of the patient/population/problem, intervention, comparison, and outcomes of the selected clinical questions

	Patient, population, problem	Intervention	Comparison	Outcome
CQ1	Adult patients diagnosed with or suspected of having infection, bacteremia, or sepsis	Balanced crystalloid administration	0.9% sodium chloride administration	Short-term mortality (28-day mortality or 30-day mortality)<br>Renal replacement therapy<br>Hyperkalemia<br>Mechanical ventilation<br>Vasopressor<br>AKI<br>Length of ICU stay
CQ2	Adult patients with sepsis	Targeting a higher mean arterial pressure	Targeting a lower mean arterial pressure	Short-term mortality (Approximately 28-day mortality)<br>Severe adverse events (cardiovascular events, bowel ischemia, limb necrosis)<br>Renal replacement therapy<br>Length of ICU stay
CQ3	Adult patients with sepsis presenting with severe metabolic acidosis	Sodium bicarbonate administration	No sodium bicarbonate administration	Short-term mortality (28-day mortality or 30-day mortality)<br>Organ failure<br>Renal replacement therapy<br>Length of vasopressor use or vasopressor-free days

				Length of ICU stay<br>Electrolyte abnormalities requiring therapeutic intervention
CQ4	Adult patients with sepsis or septic shock	Usual care with at least one of the following tissue perfusion parameters: lactate/lactate clearance, capillary refill time, $ScvO_2$/SvO_2, and P(v-a) CO2/C (a-v) O_2.	Usual care with different parameters mentioned in the interventional group, or standard care without the utilization of any specific tissue perfusion parameters	Mortality (up to 90 days)<br>Length of ICU stay<br>ICU mortality<br>Ventilator-free days
CQ5	Adult patients with sepsis, sepsis-induced hypotension, or septic shock	Restrictive fluid management, which aims to reduce the amount of fluid therapy for up to 24 h	Conventional fluid management or non-restrictive fluid management defined by authors	90-day mortality<br>Short-term mortality<br>Severe AKI or renal replacement therapy<br>Ventilator-free days<br>Severe adverse events<br>Vasopressor-free days<br>Lung edema<br>Amount of fluid administration

CQ, clinical question; AKI, acute kidney injury; ICU, intensive care unit.

eTable 2. The list of unidentified studies using the semi-automated citation screening software for the qualitative analysis

	Year	Journal	Title
CQ1	2019	American Journal of Respiratory and Critical Care Medicine	Balanced crystalloids versus saline in sepsis. A secondary analysis of the SMART Clinical Trial
CQ3	2023	Critical Care Medicine	Long-term outcome of severe metabolic acidemia in ICU patients, a BICAR-ICU trial post hoc analysis
CQ4	1995	New England Journal of Medicine	A trial of goal-oriented hemodynamic therapy in critically ill patients. SvO2 Collaborative Group

BICAR-ICU, sodium bicarbonate therapy for patients with severe metabolic acidaemia in the intensive care unit; CQ, clinical question; ICU, intensive care unit; SMART, isotonic solutions and major adverse renal events trial; SvO2, mixed venous oxygen saturation.

eTable 3. Comparison of citation screening time for 100 studies per person between the semi-automated and conventional methods

	Semi-automated citation screening			Conventional citation screening		
	Median (min)	Min (min)	Max (min)	Median (min)	Min (min)	Max (min)
CQ1	0.8	0.5	1.1	14.7	13.1	18.7
CQ2	1.2	1.0	1.4	11.9	10.0	31.1
CQ3	3.4	2.3	4.5	16.3	7.9	31.2
CQ4	1.3	1.2	1.3	15.8	13.1	27.7
CQ5	1.5	1.1	1.9	11.7	10.5	17.1

CQ, clinical question

eTable 4. Accuracy of the semi-automated citation screening software with an increased number of tagged references

A. Primary analysis

	TN	FP	FN	TP	Sensitivity (95% CI)	Specificity (95% CI)	Positive predictive value (95% CI)
Original setting (100 references)							
CQ1	5,493	29	85	27	0.241 (0.171–0.328)	0.995 (0.993–0.996)	0.482 (0.357–0.610)
Modified setting (200 references)							
CQ1	5,491	31	56	56	0.500 (0.409–0.591)	0.994 (0.992–0.996)	0.643 (0.539–0.736)

B. Secondary analysis[a]

	TN	FP	FN	TP	Sensitivity	Specificity	Positive predictive value
Semi-automated citation screening (100 references)							
CQ1	5,577	49	1	7	0.875 (0.529–0.994)	0.991 (0.989–0.993)	0.125 (0.062–0.236)
Semi-automated citation screening (200 references)							
CQ1	5,547	79	0	8	1.000 (0.676–1.000)	0.986 (0.983–0.989)	0.092 (0.047–0.171)

CI, confidence interval; CQ, clinical question; FN, false negative; FP, false positive; TN, true negative; TP, true positive.

The sensitivity, specificity, and positive predictive value are presented with 95% confidence intervals.

[a] The final list of the included studies for the qualitative analysis was set as the standard reference.

eFigure 1

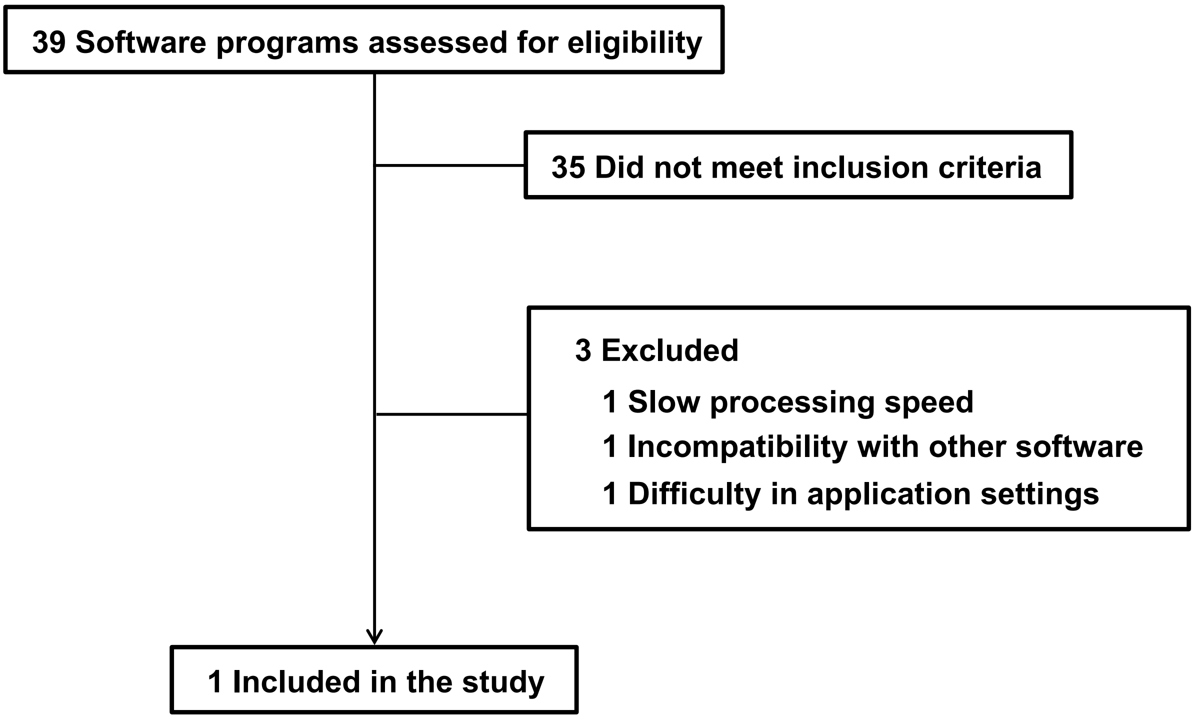
39 Software programs assessed for eligibility
35 Did not meet inclusion criteria
3 Excluded
1 Slow processing speed
1 Incompatibility with other software
1 Difficulty in application settings
1 Included in the study

eFigure 2

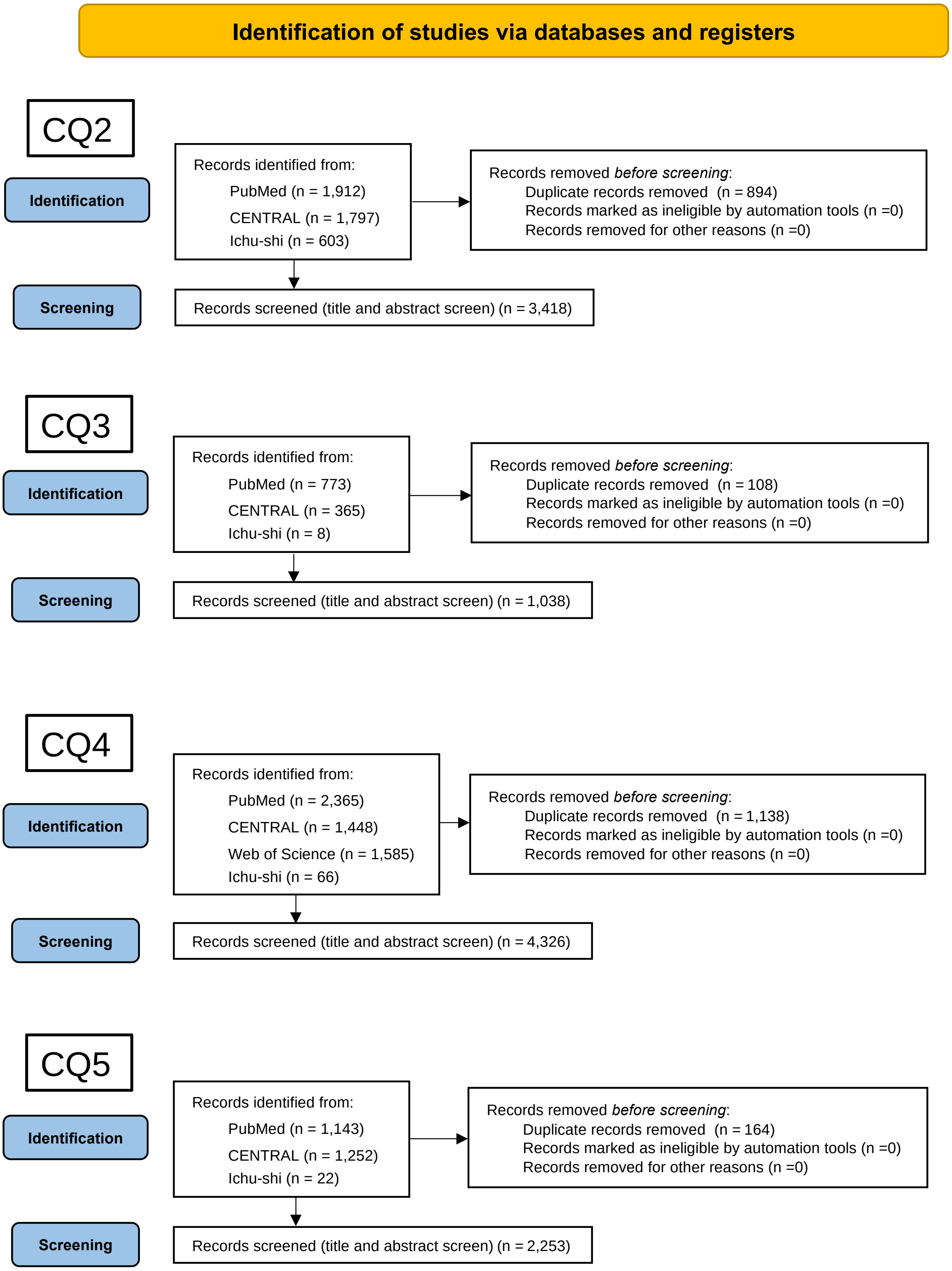
Identification of studies via databases and registers
CQ2
Identification
Records identified from:
PubMed (n = 1,912)
CENTRAL (n = 1,797)
Ichu-shi (n = 603)
Records removed *before screening*:
Duplicate records removed (n = 894)
Records marked as ineligible by automation tools (n =0)
Records removed for other reasons (n =0)
Screening
Records screened (title and abstract screen) (n = 3,418)
CQ3
Identification
Records identified from:
PubMed (n = 773)
CENTRAL (n = 365)
Ichu-shi (n = 8)
Records removed *before screening*:
Duplicate records removed (n = 108)
Records marked as ineligible by automation tools (n =0)
Records removed for other reasons (n =0)
Screening
Records screened (title and abstract screen) (n = 1,038)
CQ4
Identification
Records identified from:
PubMed (n = 2,365)
CENTRAL (n = 1,448)
Web of Science (n = 1,585)
Ichu-shi (n = 66)
Records removed *before screening*:
Duplicate records removed (n = 1,138)
Records marked as ineligible by automation tools (n =0)
Records removed for other reasons (n =0)
Screening
Records screened (title and abstract screen) (n = 4,326)
CQ5
Identification
Records identified from:
PubMed (n = 1,143)
CENTRAL (n = 1,252)
Ichu-shi (n = 22)
Records removed *before screening*:
Duplicate records removed (n = 164)
Records marked as ineligible by automation tools (n =0)
Records removed for other reasons (n =0)
Screening
Records screened (title and abstract screen) (n = 2,253)
